# Reestablishing Neuronal Networks in the Aged Brain by Stem Cell Factor and Granulocyte-Colony Stimulating Factor in a Mouse Model of Chronic Stroke

**DOI:** 10.1371/journal.pone.0064684

**Published:** 2013-06-04

**Authors:** Lili Cui, Sasidhar R. Murikinati, Dongliang Wang, Xiangjian Zhang, Wei-Ming Duan, Li-Ru Zhao

**Affiliations:** 1 Department of Neurosurgery, State University of New York Upstate Medical University, Syracuse, New York, United States of America; 2 Department of Neurology, Louisiana State University Health Sciences Center, Shreveport, Louisiana, United States of America; 3 Department of Cellular Biology and Anatomy, Louisiana State University Health Sciences Center, Shreveport, Louisiana, United States of America; 4 Department of Public Health and Preventive Medicine, State University of New York Upstate Medical University, Syracuse, New York, United States of America; 5 Department of Neurology, Second Hospital of Hebei Medical University, Shijiazhuang, Hebei, China; 6 Department of Anatomy, Capital Medical University, Beijing, China; University of Münster, Germany

## Abstract

Stroke has a high incidence in the elderly. Stroke enters the chronic phase 3 months after initial stroke onset. Currently, there is no pharmaceutical treatment available for chronic stroke. We have demonstrated the therapeutic effects of the combination of stem cell factor (SCF) and granulocyte-colony stimulating factor (G-CSF) (SCF+G-CSF) on chronic stroke. However, it remains unclear how SCF+G-CSF repairs the brain in chronic stroke. In this study, we determined the effects of SCF+G-CSF on neuronal network remodeling in the aged brain of chronic stroke. Cortical brain ischemia was produced in 16–18 month-old transgenic mice expressing yellow fluorescent protein in layer V pyramidal neurons. SCF+G-CSF was subcutaneously injected for 7 days beginning at 3.5 months post-ischemia. Using both live brain imaging and immunohistochemistry, we observed that SCF+G-CSF increased the mushroom-type spines on the apical dendrites of layer V pyramidal neurons adjacent to the infarct cavities 2 and 6 weeks after treatment. SCF+G-CSF also augmented dendritic branches and post-synaptic density protein 95 puncta in the peri-infarct cortex 6 weeks after treatment. These data suggest that SCF+G-CSF treatment in chronic stroke remodels neural circuits in the aged brain. This study provides evidence to support the development of a new therapeutic strategy for chronic stroke.

## Introduction

Stroke is the leading cause of long-term disability in adults worldwide. Most strokes occur in elderly people over age 65 [1]. Based on the pathological progress and timing after stroke onset, a stroke is classified into three phases: acute, subacute and chronic stroke. Both metabolic changes [2] and secondary neuron loss [3] are relatively stable 3 months after stroke onset. Therefore, 3 months after the stroke onset is considered the chronic phase of stroke. Pharmaceutical treatment for chronic stroke is currently not available.

Stem cell factor (SCF) and granulocyte-colony stimulating factor (G-CSF) are two essential members in the hematopoietic growth factor family that regulate bone marrow stem cell proliferation, differentiation and mobilization [4], [5]. However, increasing evidence has suggested that SCF and G-CSF also play roles in the central nervous system. SCF promotes neurite outgrowth [6], and mice with mutations of SCF [7] or SCF receptors [8] show impairments in long-term potentiation (LTP) and the spatial learning and memory. G-CSF deficient mice display cognitive impairment, LTP reduction, and poor neuronal networks in the hippocampus [9]. Interestingly, both SCF and G-CSF can pass through the blood-brain barrier [10], suggesting a potential role of SCF and G-CSF in regulating neuronal plasticity in the brain. We have demonstrated that in the phase of chronic stroke a systemic administration of SCF+G-CSF but not SCF and G-CSF alone induces a stable and long-term functional improvement accompanied by an enhanced neuronal activity in the cortex of lesioned hemisphere [11]. However, the mechanism underlying SCF+G-CSF-induced brain repair during chronic stroke remains unclear.

Functional improvement in stroke survivors is associated with neuronal network rewiring in the intact brain regions that have anatomical connections to the damaged neurons in the infarct area [12]. Therapeutic interventions that enhance the neuronal network regeneration may therefore improve outcomes in chronic stroke. Dendritic spines, the small membranous protrusions extending from the dendritic shafts, are the postsynaptic sites of neuronal connections receiving the majority of excitatory inputs of the postsynaptic neurons [13]. Dendritic spines in the peri-infarct cortex are highly dynamic after stroke [14], [15]. It has been proposed that the generation of new synaptic connections within the peri-infract brain region is involved in functional recovery after stroke [16].

The aim of this study was to determine the effects of SCF+G-CSF treatment on dendritic spine formation and dendritic branching in the peri-infarct cortex of aged brain in chronic stroke.

## Materials and Methods

The experiments have been carried out in accordance with the National Institutes of Health Guide for the Care and Use of Laboratory Animals in the United States. All experimental procedures have been approved by the Animal Care and Use Committees of State University of New York Upstate Medical University (CHUA#338) and Louisiana State University Health Sciences Center (P-12-020).

### Transgenic Mice

Aged, male transgenic mice (16–18 months old) expressing yellow fluorescent protein (YFP) driven by Thy1 promoter (H-line) (Thy1-YFPH) [17] (The Jackson Laboratory) were used in this study. In these Thy1-YFPH mice, pyramidal neurons in the layer V are specifically tagged with YFP, and the YFP is expressed in the somata, axons, dendrites and dendritic spines [17]. Mice were housed in a standard laboratory environment (12 h light/12 h dark regime at 26°C) and were given ad libitum access to food and water.

### Animal Model of Stroke

Focal cerebral cortical ischemia (stroke) was conducted by permanent occlusion of both the middle cerebral artery (MCA) and common carotid artery (CCA). Animals were anesthetized with an intraperitoneal injection of Avertin (240 mg/kg; Sigma-Aldrich). Body temperature was monitored and maintained at 37°C during the surgery by use of a heating pad coupled to a temperature regulator. A midline incision was made on the neck, and the right CCA was ligated with a 3–0 silk surgical suture. Another incision was then made between the right ear and eye. After a craniotomy was conducted using a high-speed dental drill, the MCA was coagulated with a cauterizer (Bovie® AAron medical).

### Experimental Design

**Figure 1:**
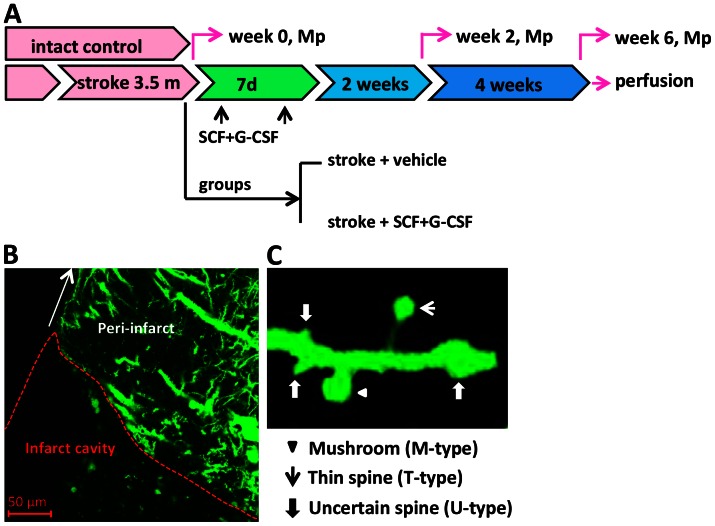
Experimental design and live brain imaging. **(A) Schematic diagram of experimental design of this study.** At 3.5 months after induction of focal brain ischemia (stroke), mice were randomized to receive subcutaneous injection of vehicle or SCF+G-CSF for 7 days. Intact control mice received subcutaneous injection of vehicle for 7 days. The peri-infarct cortex was scanned through a thinned-skull window with a multiphoton microscope (Mp) before treatment (week 0), 2 and 6 weeks after treatment. At the end of the imaging, mice were transcardially perfused, and brains were removed and processed for immunohistochemistry. (**B**) A representative brain section from a thy-1-YFPH mouse with chronic stroke displays the peri-infarct cortex where the multiphoton imaging is performed. (**C**) A representative apical dendrite of the layer V pyramidal neuron expressing YFP illustrates different spine shapes including a mushroom type spine, a thin type spine and uncertain type spines. A mushroom type spine (M-type) with a large spine head and a thick neck. A thin type spine (T-type) with a small head and a narrow neck. Uncertain type spines (U-type) with undetectable heads and an equal diameter in the spine tip and stem or varicosity protuberance from the dendritic shaft.

At 3.5 months after brain ischemia, mice were randomly assigned into two groups: 1) stroke+vehicle, in which animals with stroke received equal volumes of saline and 5% dextrose (n = 6); and 2) stroke+SCF+G-CSF, in which animals with stroke received recombinant mouse SCF (200 µg/kg/day; PeproTech) and recombinant human G-CSF (50 µg/kg/day; Amgen) (n = 6). SCF, G-CSF, and vehicle solution were subcutaneously injected for 7 days beginning at 3.5 months after stroke. A third group of age-matched mice without stroke served as an intact control, in which animals received subcutaneous injection of equal volumes of saline and 5% dextrose for 7 days (n = 3). The day before the vehicle and SCF+G-CSF treatment was defined as week 0. The brains of the mice in the three groups were scanned with a multi-photon microscope through a thinned-skull window before treatment (week 0), 2 and 6 weeks after treatment (Figure 1A).

### Thinned-skull Window

The surgical procedures for preparing a thinned-skull window have been described elsewhere [18]. Briefly, after being anesthetized with Avertin, mice were immobilized on a stereotaxic apparatus (David Kopf Instruments). An incision was made in the midline on the scalp, and an area up to 3 mm×3 mm over the infarct cavity was thinned using a high-speed micro drill (Hager & Meisinger Gmbh). To avoid damaging the underlying cortex by friction-induced heat, the thinning of the skull was only performed for a few seconds at a time, and the skull was constantly wetted with cool, sterile saline. The thinning was completed by gently scraping the skull with a microsurgical blade until the pial blood vessels were clearly visible under the microscope.

### Multiphoton Microscope Imaging

Before treatment (week 0), and at 2 and 6 weeks after treatment, the apical dendritic tufts of the YFP-labeled layer V pyramidal neurons distributing in the layer I were repeatedly imaged from different fields in the same mice using a Zeiss multiphoton microscope (Zeiss LSM 510 NLO). At each time point, a region of the sensorimotor cortex approximately 1 mm from the infarct cavity border was selected (Figure 1B). The parameters for the live brain imaging with the multiphoton microscope were set up as the follows: three sites per brain, several dendritic arbors for each site, and up to 100 µm below the pial surface. YFP was excited with a multiphoton near*-*infrared pulsed laser (Chameleon, Coherent) at 920 nm, and images were acquired using a 40× water-immersion objective (0.8 numerical aperture) with 1 µm- apart high-resolution z-stacks and 512×512 pixel resolution. Throughout the imaging process, anesthesia was performed with Avertin, and body temperature was maintained with a heating pad.

The number of dendritic spines on the selected dendrites (2∼4 segments per field, 20–30 µm for each segment) from three different fields in sensorimotor cortex was quantified using Zeiss LSM-5 examiner software. In order to make a clear classification of a spine and also to prevent the repetitive counts of same spines, the scroll bar on the side of the serial images from z-stacks was drawn up and down. Measurements of spine head size were performed by drawing a line on the widest part of the spine heads from the z-stacks. The percentage and the spine density (number of spines per 10 µm of dendrite) of the different types of spines were then calculated. In this study, dendritic spines were categorized into three types based on their morphological features: 1) mushroom spine (M-type) [18], [20], 2) thin spine (T-type) [20] and 3) uncertain spine (U-type) (Figure 1C). The mushroom spine has a large, well-defined spine head (>0.8 µm) and a thin neck. The thin spine has a smaller head (<0.8 µm), a thinner and longer neck than the mushroom spines. The uncertain spine has a poorly-defined spine head and a thickened or no distinguishable spine neck. Some protrusions from the dendritic shafts with varicosity and swelling were also classified as uncertain spines. The uncertain type spines include the stubby type spines [19], the nubby type and dimple type spines as described by others [20].

### Immunohistochemistry

At 6 weeks after the final scanning, mice were transcardially perfused with phosphate buffered saline (PBS) followed by 4% buffered formaldehyde. One of the mice in the stroke control group died after week 6 imaging. The brain of this mouse was excluded for immunohistochemistry because it was not well fixed. In addition, two more age-matched intact controls were added for immunohistochemistry. The brains were post-fixated in 4% formaldehyde, cryoprotected in 30% sucrose in PBS, and sectioned into 30 µm-thickness sections. The brain sections were kept in anti-freeze medium and stored at −20°C until used for immunohistochemistry. Adjacent brain sections (3–5 sections/brain) through the infarct cavities were processed for immunohistochemistry with free-floating section method. Nonspecific binding was blocked with 5% normal goat serum diluted by 1% bovine serum albumin (IgG free; Jackson ImmunoResearch Labs) and 0.25% Triton X-100 (Sigma) in PBS for 45 min at room temperature. Brain sections were then incubated with primary antibodies overnight at 4°C. The primary antibodies used in the study included a monoclonal mouse anti-microtubule associated protein 2 (MAP2, 1∶500, Sigma) and a monoclonal mouse anti-post synaptic density protein 95 (PSD-95, 1∶200, Sigma). For each setting of immunostaining, brain sections without incubation of primary antibodies served as negative controls. After washing with PBS, the sections were exposed with DyLight 549-conjugated goat anti-mouse secondary antibody (1∶400, Jackson ImmunoResearch) for 2h in the dark at room temperature, and mounted with ProLong Gold Antifade reagent (Life Technologies).

In each section, eight consecutive 56 µm×56 µm fields beginning from the border of infarct cavities were imaged in the layer I of the cortex outside the infarct cavities for MAP2 immunostaining. For detecting PSD-95 positive staining, two fields in layer I to layer III cortical regions surrounding the infarct cavities (within 500 µm from infarct cavity) were scanned. All images were captured using a Zeiss confocal microscopy (LSM 510 NLO) with 1 µm-interval- high-resolution z-stacks and 512×512 pixel resolution. The z-stacks images for MAP2 and PSD95 were then reconstructed into 3-dimensional-reconstruction images with the Zeiss LSM 510 software. To reduce the noise, a median filter (size X axis: 3, Y axis: 3, Z axis: 1) was applied to all images before reconstruction. Thereafter, the reconstructed images were exported and analyzed using Image J software. For MAP2 quantification, the mean optical density of each picture was automatically calculated. To reduce the influences induced by immunoreactivity of the antibodies, the mean optical density of MAP2 were subtracted by the background optical density for each picture. For PSD 95 quantification, the number of PSD-95-labled dendritic puncta with a threshold of 30 pixels was automatically obtained by Image J software.

### Statistical Analysis

The three-group comparison was examined using one-way or two-way ANOVA followed by Bonferroni/Dunn correction. Comparison between the two groups was analyzed with an unpaired student’s t-test. All data are expressed as mean ± S.E.M. The statistical significance level was set at P<0.05. The experiments were performed in a randomized and blinded manner.

## Results

### The Effects of SCF Plus G-CSF on Total Apical Spine Density in the Peri-infarct Cortex of the Aged Brain During Chronic Stroke

**Figure 2:**
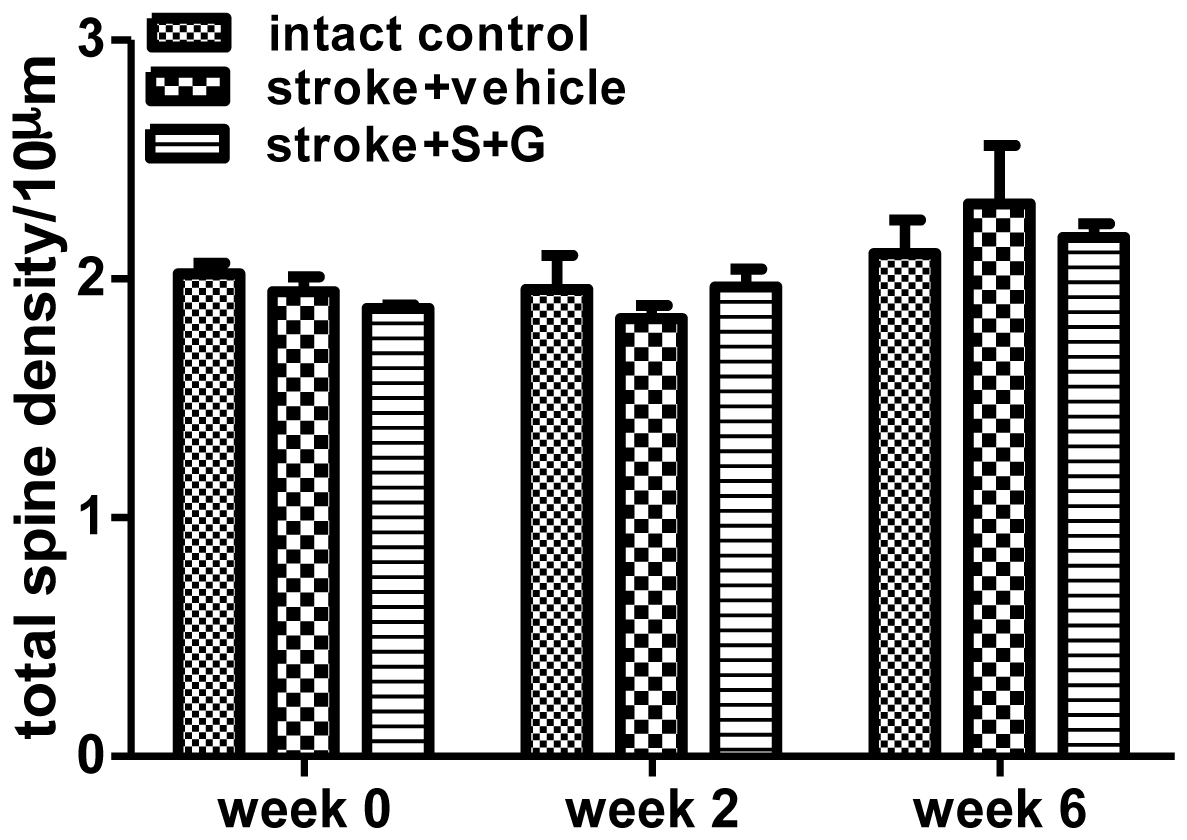
The effect of SCF+G-CSF on total spine density of the apical dendrites of the layer V pyramidal neurons in the peri-infarct cortex of aged brain in chronic stroke. Live brain imaging in the cortex adjacent to the infarct cavities or the corresponding cortex of intact mice was performed before treatment (week 0), 2 and 6 weeks after treatment. Note that there is no significant difference in total spine density of aged brain in chronic stroke among the intact controls (n = 3), stroke with vehicle treatment (n = 6), and stroke with SCF+G-CSF treatment (n = 6) neither before nor after treatment. Apical spine density: number of spines per 10 µm dendrite length. Mean ± S.E.M.

To determine the extent to which SCF+G-CSF affects total dendritic spine density in the aged brain during chronic stroke, we imaged and analyzed the layer I apical dendritic spines of layer V pyramidal neurons in the cortex adjacent to the infarct cavity before treatment (week 0), 2 and 6 weeks after treatment in the same mice. At week 0, there were no differences in total apical spine density among the groups of the intact controls, stroke vehicle controls, and stroke SCF+G-CSF treatment (Figure 2) (one- way ANOVA: F(2,6) = 2.57, P = 0.16), indicating that the total number of spines in the peri-infarct cortex of the aged brain is not affected in the chronic stroke. After the first scanning at week 0, stroke mice were treated with either SCF+G-CSF or an equal volume of vehicle for 7 days, and the brain was scanned again using a multiphoton microscope at 2 and 6 weeks after the final injection. We found that total spine density did not show significant differences among the groups of intact control, stroke vehicle control and stroke SCF+G-CSF treatment either 2 weeks (one- way ANOVA: F(2,6) = 0.53, P = 0.60) or 6 weeks (one- way ANOVA: F(2,6) = 0.40, P = 0.69) after treatment (Figure 2). In addition, the total spine density was not affected by the interaction between the groups and selected time points, and no significant changes were seen over the period of 6 weeks (two-way ANOVA: F(4,12) = 0.884, P = 0.50) (Figure 2). Furthermore, the temporal changes of total spine density during imaging at each selected-time point did not show significant variation among the 3 groups (data not shown). These data suggest that SCF+G-CSF treatment during chronic stroke does not influence the total spine density.

### Morphological Changes of Dendritic Spines are Seen in the Aged Brains of Chronic Stroke Mice Before Treatment

**Figure 3:**
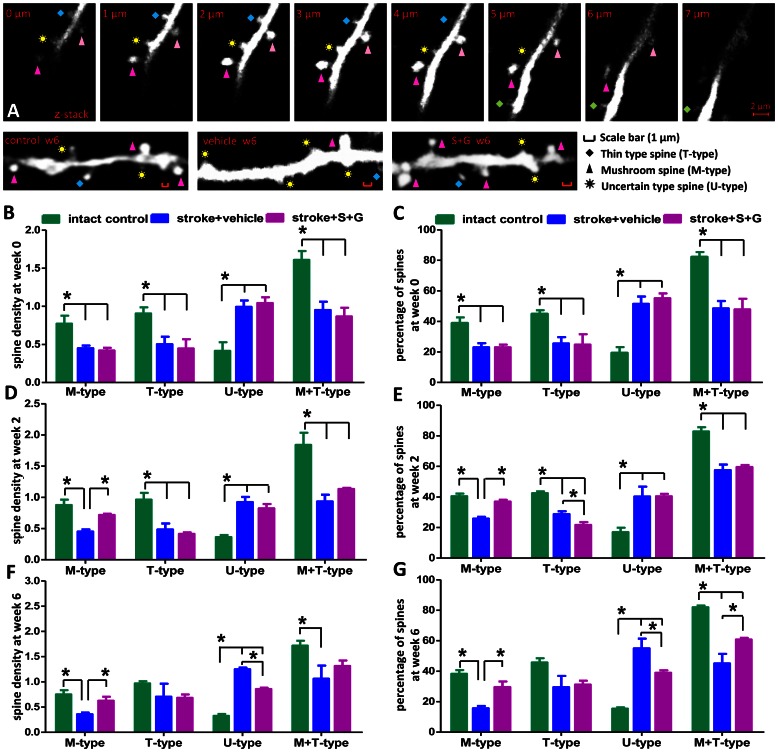
Dynamics of the apical dendritic spines of layer V pyramidal neurons in the peri-infarct cortex of aged brain before SCF+G-CSF treatment (week 0), 2 and 6 weeks after treatment in the phase of chronic stroke by live brain imaging. (**A**) Upper panels: Representative Z-stack images with 1-µm intervals display the three types of spines–mushroom type, thin type and uncertain type spines. Lower panels: Representative live brain images show the apical dendritic spines in the right cortex of an intact control or in the peri-infarct cortex of stroke mice with vehicle injection or stroke mice with SCF+G-CSF (S+G) treatment at 6 weeks (6 w) post-treatment. Scale bars (red), 1 µm. (**B and C**) Quantification of apical spine density (**B**) and the percentage of different types of spines (**C**) in the peri-infarct cortex before treatment (week 0). (**D and E**) Quantification of apical spine density (**D**) and the percentage of different subtypes of spines (**E**) in the peri-infarct cortex 2 weeks after SCF+G-CSF treatment. (**F and G**) Quantification of apical spine density (**F**) and percentage of different subtypes of spines (**G**) in the peri-infarct cortex 6 weeks after SCF+G-CSF treatment in the aged brain of chronic stroke. *P<0.05. Intact control, n = 3; stroke+vehicle, n = 6; stroke+S+G, n = 6. Mean ± S.E.M. M-type, mushroom type spine; T-type, thin type spine; U-type, uncertain type spine. Apical spine density: number of spines per 10 µm dendrite length.

To determine whether there are any morphological changes in the apical dendritic spines of the layer V pyramidal neurons surrounding infarct cavities in the aged brain of chronic stroke, we quantified mushroom type (M-type), thin type (T-type) and uncertain type (U-type) spines (Figure 1A) before treatment (week 0). As shown in Figure 3B and C, the most abundant spine types in the brain of the intact control group were mushroom and thin spines, while uncertain spines only constituted a small portion of the total spines. However, mushroom type, thin type, and mushroom+thin (M+T)-type spines were significantly decreased in the peri-infarct cortex of the aged chronic stroke brain at week 0 as compared with those of the intact control mice (Figure 3B and C, P<0.05) (one- way ANOVA: M-type, F(2,6) = 8.59, P = 0.02; T-type, F(2,6) = 6.10, P = 0.04; M+T-type, F(2,6) = 12.96, P = 0.007). In addition, a significant increase in uncertain spines was also seen in all chronic stroke mice that would receive vehicle or SCF+G-CSF treatment later (Figure 3B and C, P<0.05) (one- way ANOVA: F(2,6) = 14.23, P = 0.005; stroke groups vs. intact control group). No significant differences in each type of spines in the peri-infarct cortex were observed between the vehicle controls and SCF+G-CSF group at week 0 (Figure 3B and C, P>0.05). Mushroom and thin spines contribute to building up synaptic connections with other neurons, and uncertain spines are often seen in the brain of neurodegenerative diseases [21]. These data therefore suggest that the morphological changes in the apical dendritic spines of the layer V pyramidal neurons surrounding the cortical infarct cavities in the chronic phase are cortical infarct-related. This may imply that the apical dendritic spines of the layer V pyramidal neurons in the cortex adjacent to the infarct cavities undergo degeneration because they lose synaptic connections with the neurons that have been lost due to the ischemic damage in the early stage of stroke.

### SCF Plus G-CSF Increases Mushroom Spines in the Peri-infarct Cortex of the Aged Brain 2 Weeks After Treatment During Chronic Stroke

Next we wanted to determine the effects of SCF+G-CSF on the modification of spine types of layer V neurons in the peri-infarct cortex of an aged brain 2 weeks after treatment. In vehicle-treated stroke mice, we found the same results as seen at week 0 that the mushroom spines, thin spines and M+T spines were significantly decreased, and that the uncertain spines were significantly increased 2 weeks after treatment as compared with the intact controls (Figure 3D and E, P<0.05) (one- way ANOVA: M-type, F(2,6) = 16.75, P = 0.004; T-type, F(2,6) = 18.76, P = 0.003; M+T-type, F(2,6) = 16.36, P = 0.004; U-type, F(2,6) = 18.37, P = 0.003). However, the mushroom spines in the brains of SCF+G-CSF-treated stroke mice were significantly increased as compared with the vehicle controls (Figure 3D and E, P<0.05). Interestingly, the percentage of thin spines was decreased slightly by SCF+G-CSF 2 weeks after treatment in comparison with the stroke vehicle controls (Figure 3E, P<0.05). At 2 weeks post-treatment, the uncertain spines of SCF+G-CSF group were still greater than the intact controls (Figure 3D and E, P<0.05), and no differences were noted as compared with the stroke vehicle controls. Previous studies have shown that only mushroom-type spines can build strong and long-lasting synaptic connections with other neurons [22]. These data therefore suggest that SCF+G-CSF treatment in chronic stroke promotes a re-establishment of synaptic networks with other neurons in the peri-infarct cortex of the aged brain.

### SCF Plus G-CSF Increases Mushroom Spines and Decreases Uncertain Spines in the Peri-infarct Cortex of the Aged Brain 6 Weeks After Treatment During Chronic Stroke

To further determine whether SCF+G-CSF treatment has a long-term effect on the modification of spine morphology in the aged brain of chronic stroke, the apical dendritic tufts of layer V pyramidal neurons in the peri-infarct cortex were scanned again in the same mice 6 weeks after SCF+G-CSF treatment. In line with the findings of week 0 and 2, at 6 weeks post-treatment we observed that only the stroke vehicle controls still showed a significant decrease in mushroom spines and M+T spines and a significant increase in uncertain type spines as compared with the intact controls (Figure 3F and G, P<0.05) (one- way ANOVA: M-type, F(2,6) = 8.75, P = 0.02; M+T-type, F(2,6) = 23.24, P = 0.002; U-type, F(2,6) = 198.37, P<0.0001). By contrast, SCF+G-CSF-treated mice displayed the same results as observed at 2 weeks post-treatment that the mushroom spines were significantly increased 6 weeks after treatment when compared to the stroke vehicle controls (Figure 3A, F and G, P<0.05). In addition, the percentage of M+T spines in SCF+G-CSF-treated mice was also significantly greater than those in the stroke vehicle controls (Figure 3F and G, P<0.05). Remarkably, the uncertain type spines were significantly reduced by SCF+G-CSF at 6 weeks post-treatment when compared to the stroke vehicle controls (Figure 3F and G, P<0.05). The thin spines were found to have no difference among the groups of intact control, stroke vehicle control and stroke SCF+G-CSF treatment at 6 weeks post-treatment (Figure 3F and G, P>0.05) (one- way ANOVA: T-type, F(2,6) = 1.05, P = 0.41). Taken together, SCF+G-CSF treatment in chronic stroke induces a steady increase in mushroom spines (at 2 and 6 weeks) and a delayed reduction of uncertain spines (at 6 weeks) in the peri-infarct cortex of aged brain, suggesting a long-term effect of SCF+G-CSF on re-establishing synaptic networks in the peri-infarct cortex of the aged brain in chronic stroke.

### SCF Plus G-CSF Treatment During Chronic Stroke Increases Apical Dendritic Branching in the Peri-infarct Cortex of the Aged Brain

**Figure 4:**
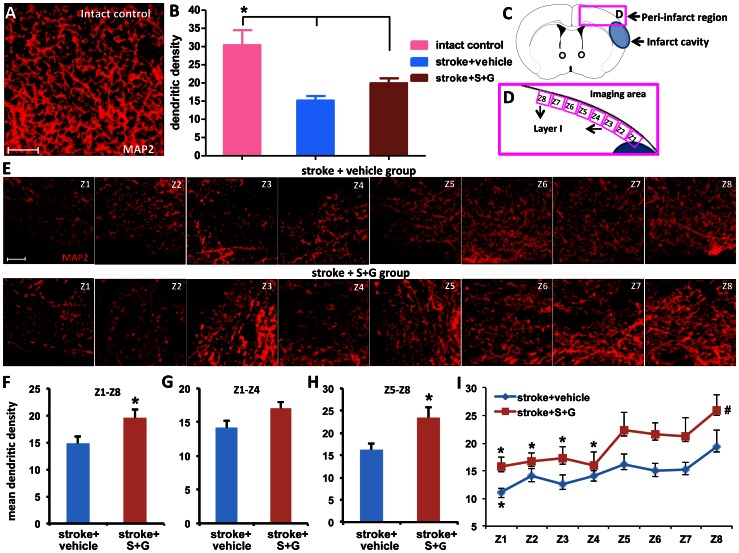
SCF+G-CSF treatment in chronic stroke increases dendritic density in layer I cortex outside the infarct cavities in the aged brain 6 weeks after treatment. (**A**) A representative 3-dimensional (3-D) projected image of the apical dendrites in the intact brain. Scale bar, 10 µm. (**B**) Quantification of apical dendritic density in corresponding regions in the brains of intact controls and the peri-infarct cortex 6 weeks after treatment. *p<0.05. (**C** and **D**) Schematic graphs indicate the 8-image fields that are acquired in layer I of the cortex outside the infarct cavity. (**E**) Representative 3-D projected images taken from the 8 fields of the apical dendrites in layer I cortex outside the infarct cavity in both stroke+vehicle controls and stroke+ S+G group 6 weeks after treatment. Scale bar, 10 µm. (**F–H**) Quantification of the apical dendritic density in the total region of Z1–Z8, the proximal region (Z1–Z4) and distal region (Z5–Z8) 6 weeks after treatment, respectively. *P<0.05. (**I**) Spatial distribution of apical dendritic density in the cortex outside of the infarct cavities 6 weeks after treatment. *P<0.05 vs. Z8, #P<0.05 vs. stroke controls (Z1-Z8). Intact control, n = 5; stroke+vehicle, n = 5; stroke+S+G, n = 6. Mean ± S.E.M.

To examine the effects of SCF+G-CSF treatment on dendritic branching, brain samples were collected at the end of live brain imaging (6 weeks after treatment), and dendritic density was determined by MAP2-labeled dendrites using immunohistochemistry and confocal imaging. We observed that the dendritic branches in the layer I frontal cortex of the lesion side brain (right brain) was significantly reduced in the stroke mice as compared to the intact mice (Figure 4A–E) (one- way ANOVA: F(2,13) = 8.91, P = 0.004. Stroke+vehicle vs. control, P = 0.001; stroke+S+G vs. control, P = 0.01). In addition, when we examined the density of MAP2-labeled dendrites in the regions proximal (Figure 4G, Z1∼Z4) and distal (Figure 4H, Z5∼Z8) to the infarct cavities, we found that the apical dendritic density varied according to the dendrites’ distance from the infarct border in the aged brain of chronic stroke (Figure 4I). Along the layer I frontal cortex outside the infarct cavities, dendritic density was lower in regions proximal to the infarct cavities compared to the distal regions in the aged brain of chronic stroke (Figure 4I, P<0.05). SCF+G-CSF treatment, however, induced a significant increase in apical dendritic density in the distal region and a trend toward increase in the proximal region of the frontal cortex (Figure 4F and G, P<0.05). In the parietal cortex surrounding the infarct cavity, the apical dendritic density was significantly increased by SCF+G-CSF (Figure S1).

In addition, the apical dendritic density in the whole region (Z1-Z8) was also significantly increased by SCF+G-CSF 6 weeks after treatment (Figure 4B, 4F and 4I, Z1∼Z8, P<0.05). These data suggest that SCF+G-CSF treatment in the phase of chronic stroke enhances neuronal network remodeling in the cortex outside the infarct cavities in the aged brain.

In the contralateral cortex, however, the MAP2-labeled dendrites did not show differences between the two stroke groups: the vehicle controls and SCF+G-CSF-treated (Figure S2), suggesting the reparative effects of SCF+G-CSF on chronic stroke.

### SCF Plus G-CSF Treatment During Chronic Stroke Increases Post-synaptic Density Protein 95 (PSD-95) Puncta in the Peri-infarct Cortex of Aged Brain

**Figure 5:**
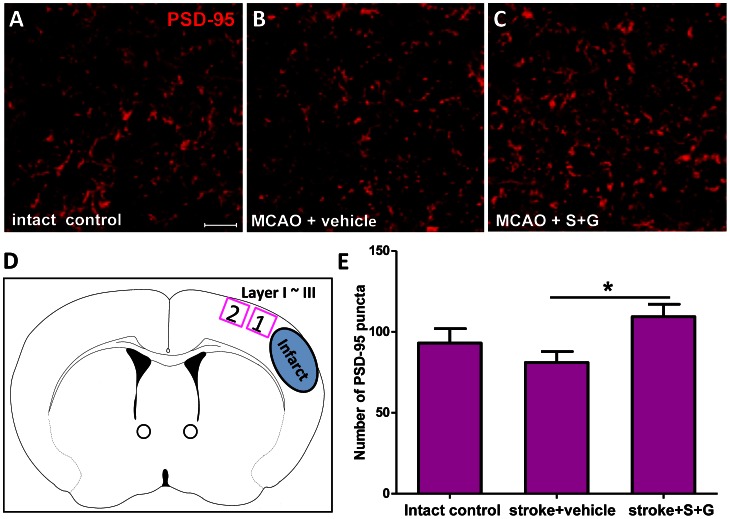
SCF+G-CSF treatment during chronic stroke increases PSD-95 puncta in the peri-infarct cortex of aged brain 6 weeks after treatment. (**A**–**C**) Three-dimensional projection of PSD-95 puncta detected by immunohistochemistry in layer I∼III of the corresponding cortex in the intact controls and the peri-infarct cortex 6 weeks after vehicle treatment or SCF+G-CSF treatment in aged chronic stroke brain. Scale bar, 20 µm. (**D**) Schematic graph showing where the images were taken. (**E**) Quantification of PSD-95 puncta (number of PSD-95 puncta per field) in the corresponding cortex of the intact controls and in the peri-infarct cortex of chronic stroke brains in aged mice 6 weeks after treatment. *P<0.05. Intact control, n = 5; stroke+vehicle, n = 5; stroke+S+G, n = 6. Mean ± S.E.M.

We then sought to determine whether SCF+G-CSF treatment during chronic stroke enhances the characteristics of synapses in the aged brain. Substantial evidence has supported that PSD-95 plays a critical role in the regulation of excitatory postsynaptic function and strength [23]. We therefore quantified PSD-95 puncta in layer I∼III of the peri-infarct cortex using immunohistochemistry 6 weeks after treatment (Figure 5A–D). Three-dimensional confocal images of PSD-95 positive puncta were analyzed using Image J software. We found that SCF+G-CSF treatment during chronic stroke resulted in a 35% increase in PSD-95 puncta in the peri-infarct cortex of the aged brain when compared to the stroke vehicle group (Figure 5E) (vehicle control vs. SCF+G-CSF: 81.01±6.84 vs. 109.44±7.93, P<0.05). This result provides additional evidence supporting that SCF+G-CSF treatment during chronic stroke increases the formation of synaptic networks in the peri-infarct cortex of aged brain.

## Discussion

In this study we have determined the effects of SCF+G-CSF on neuronal network remodeling in the peri-infarct cortex of the aged brain in chronic stroke. We have revealed that 1) mushroom and thin type spines are reduced, and uncertain type spines are increased in the chronic stroke brain, 2) SCF+G-CSF increases the mushroom spines and reduces the uncertain type spines, and 3) dendritic branching and synapse formation are enhanced by SCF+G-CSF. These findings suggest that SCF+G-SCF treatment in chronic stroke can promote dendritic remodeling and re-establish neuronal connections in the peri-infarct cortex of an aged brain. These observations are consistent with a previous finding that the SCF+G-SCF-induced rewiring neuronal networks occur in the cortex of lesion-side brain in chronic stroke, and the neuronal network remodeling in the peri-infarct cortex may contribute to the stable improvement of sensorimotor function caused by SCF+G-CSF treatment in chronic stroke [11].

Rewiring neuronal networks in the peri-infarct cortex by forming new synaptic connections has been documented to contribute to functional rehabilitation after stroke [24]. Dendritic spines are the postsynaptic membranes building up synaptic connections with the axonal terminals of excitatory neurons; therefore, dendritic spines can serve as an index for determination of neuronal network formation.

Synaptic strength is dependent on the spine size and morphology. In the present study, we have revealed that the spine types are dynamically changed in the aged brain by the SCF+G-CSF treatment in chronic stroke. The postsynaptic density (PSD) is a protein dense specialization attached to the postsynaptic membrane. The mushroom spine has a large head containing larger and more complex PSDs [25] with high density of α-amino-3-hydroxy-5-methyl-4-isoxazole-propionic acid (AMPA) receptors [26]. The mushroom spines are also supported by endogenous organelles, such as smooth endoplasmic reticulum [27], endosomal compartments [28], polyribosomes [22] and perisynaptic astroglia [29]. These features make mushroom spines more stable and functionally stronger than thin spines. In contrast, thin spines have small PSDs only expressing N-methyl-D-aspartate (NMDA) receptors with a few AMPA receptors. The feature of thin spines makes them more flexible and enables them to change their shapes rapidly, either by enlarging and growing into large mushroom spines or by shrinking and disappearing in response to microenvironment changes [30]. The uncertain spines including stubby spines are non-synaptic protrusions representing the newborn or degenerating spines [20]. In an adult brain, the increased non-synaptic spines are the pathological hallmarks of neurodegenerative diseases, such as Alzheimer’s disease [21]. In the current study, decreased mushroom and thin spines, and increased uncertain spines have been seen in the apical dendrites of layer V pyramidal neurons in the peri-infarct cortex of aged brain in chronic stroke, suggesting that the layer V pyramidal neurons in the peri-infarct cortex are undergoing synaptic degeneration because they lose connections with the neurons that died by ischemic injury. Remarkably, SCF+G-CSF treatment beginning at 3.5 months after stroke in aged mice increases mushroom spines and decreases thin spines at 2 weeks post-treatment, and increases mushroom spines and decreases uncertain spines at 6 weeks post-treatment, suggesting that SCF+G-CSF facilitates the transition of thin spines into mushroom spines at 2 weeks post-treatment and promotes the maturation of the immature spines (uncertain spines) into mature and stable mushroom spines in a delayed time at the 6 weeks post-treatment.

A fundamental mechanism for plasticity of neural circuits is the activity-related modification of excitatory synaptic transmission. PSD-95 is a major and most stable component of the scaffolding protein PSDs at glutamatergic synaptic spines [31]. Most synaptic glutamatergic receptors, including NMDA receptors and AMPA receptors, are anchored in PSDs [32]. PSD-95 regulates synaptic AMPA receptor trafficking, retention and function [23], [33], and plays an essential role in regulating both basal excitatory transmission and synaptic plasticity. PSD-95 is therefore an impotent regulator to strengthen excitatory synapses. In our study, SCF+G-CSF induces a remarkable increase in PSD-95 puncta in the peri-infarct cortex in the aged brain of chronic stroke, suggesting the involvement of SCF+G-CSF in the enhancement of synaptic plasticity in the aged brain of chronic stroke. Together with the increased mushroom spines, the increased PSD-95 provides additional evidence in support of the enhancement of synaptic rewiring in the peri-infarct cortical neurons by SCF+G-CSF treatment in the aged brain of chronic stroke. Importantly, our recent findings reveal that SCF+G-CSF treatment in chronic stroke increases mushroom spines and PSD-95 puncta in the peri-infarct cortical neurons through the regulation of NFkB signaling (Cui et al, unpublished observation). Using functional imaging to further examine the functioning of the re-established neuronal circuits in the peri-infarct cortex after SCF+G-CSF treatment will be targeted in our future studies as the technologies of two-photon uncaging of glutamate and two-photon calcium imaging have recently been developed for detecting single synaptic spine function in live animals [34], [35].

It remains controversial whether dendrites of pyramidal neurons in the adult brain could grow more branches in contributing to functional reorganization after stroke. Brown and co-workers [36] observed a threefold increase in dendritic remodeling in the peri-infarct cortex 2 weeks after cerebral cortical ischemia, whereas Mostany and Portera-Cailliau [37] reported that apical dendritic branches in the peri-infarct cortex decreased 90 days after stroke. Here we have observed a decreased apical dendritic density in the peri-infarct cortex 5 months after cortical ischemia. Surprisingly, SCF+G-CSF treatment in chronic stroke significantly increases apical dendritic branching in the cortex outside infarct cavities, suggesting that surviving neurons in the aged brain of chronic stroke retain the capacity for dendritic remodeling and that SCF+G-CSF can enhance the dendritic branching in aged chronic stroke brain.

The re-establishment of neuronal networks in the peri-infarct cortex after SCF+G-CSF treatment is the key process for brain repair in chronic stroke. Although how SCF+G-CSF enhances mushroom spine formation and promotes dendritic branching in chronic stroke brain remains poorly understood, our recent studies suggest that NFkB signaling plays a crucial role in the SCF+G-CSF-induced neuronal plasticity. Using primary cortical neuron cultures, we observed that SCF+G-CSF promoted neurite outgrowth. In addition, SCF+G-CSF increased brain-derived neurotrophic factor (BDNF) production through NFkB signaling, and the NFkB and BDNF are required for SCF+G-CSF-induced enhancement of neurite extension (Su et al, unpublished observation). Using a mouse model of chronic stroke, we also found that blockage of NFkB signaling prevented the SCF+G-CSF-induced increase of mushroom spines and PSD-95 puncta in the peri-infarct cortex and inhibited the SCF+G-CSF-induced functional restoration (Cui et al, unpublished observation). Interestingly, BDNF has been shown to increase mushroom spine generation and reduce stubby spine formation [38]. Whether BDNF is also a key player involved in re-establishing neuronal networks in the cortex adjacent to the infarct cavity after SCF+G-CSF treatment in chronic stroke remains to be clarified in future studies.

The capability of neuronal plasticity in the undamaged brain regions to take over the function of the dead neurons has been stated to be limited according to the timing after stroke [39]. Biernaskie and co-workers (2004) [40] reported that both the functional outcome and neuronal structure changes are quite different when housing stroke rats in an enriched environment (EE) for 5 weeks initiated at 5, 14, or 30 days after brain ischemia. The best outcome was seen in the stroke rats exposed to EE at 5 days, whereas EE housing that was delayed until 30 days after stroke had no beneficial effect. In contrast to the timing limitation of the use or experience-depended neuronal remapping and functional benefits by EE, SCF+G-CSF appears to have a much broader therapeutic window. We have previously demonstrated that a systemic administration of SCF+G-CSF at 3.5 months post-stroke induces a stable and long-term functional improvement and an enhanced functional reorganization within the damaged brain [11]. The current study shows that SCF+G-CSF intervention beginning at 3.5 months post-stroke can reestablish neuronal networks in the peri-infarct cortex of the aged brain. Most importantly, this study shows that an aged brain under the condition of chronic stroke can be repaired by SCF+G-CSF. As mentioned earlier, stroke occurs more frequently in elderly people. Thus, this study sheds light on developing a new therapeutic route for chronic stroke.

In summary, a systemic administration of SCF+G-CSF in the chronic stroke promotes apical dendritic remodeling in surviving layer V pyramidal neurons adjacent to the infarct cavities of the aged brain. The prominent role of SCF+G-CSF in the enhancement of structural reorganization of neural circuits in the aged brain would provide a novel pharmacological approach to facilitate stroke rehabilitation in chronic stroke. These findings offer new insights into the role of hematopoietic growth factors in brain repair in the aged brain and also help in developing new therapeutic strategies to treat aging-related neurological disorders or neurodegenerative diseases.

## Supporting Information

Figure S1 **SCF+G-CSF treatment in chronic stroke increases dendritic density in the parietal cortex outside the infarct cavities 6 weeks after treatment in the aged brain. (A and B)** Representative images of 3-dimensional projection of MAP2-labeled dendrites in the brain of stroke with vehicle treatment (**A**) and stroke with SCF+G-CSF treatment (**B**). (**C**) A schematic graph shows where the images were taken in the parietal cortex. (**D**) Quantification of dendritic density in the layer I cortex surrounding the infarct cavities. Scale bar: 50 µm. Z1, zone 1. Z2, zone 2. **p<0.01. N = 3–4. Mean ± S.E.M.
(TIF)

Figure S2 **SCF+G-CSF treatment in chronic stroke does not affect the dendritic density in the contralateral cortex of the aged brain.** (**A–C**) Representative images of 3-dimensional projection of MAP2-labeled dendrites in the contralateral cortex (layer I) of an intact brain (**A**), the stroke brains treated with vehicle (**B**) or SCF+G-CSF (S+G) (**C**) 6 weeks after treatment. (**D**) Quantification of dendritic density in the layer I cortex contralateral to the infarct brain. N = 3–6. Mean ± S.E.M.
(TIF)

## References

[1] Roger VL, Go AS, Lloyd-Jones DM, Benjamin EJ, Berry JD, et al (2012) Heart Disease and Stroke Statistics–2012 Update: A Report From the American Heart Association. Circulation 125: 188–97. 22215894 10.1161/CIR.0b013e3182456d46

[2] Parsons MW, Li T, Barber PA, Yang Q, Darby DG, et al (2000) Combined ^1^H MR spectroscopy and diffusion-weighted MRI improves the prediction of stroke outcome. Neurology 55: 498–505. 10953180 10.1212/wnl.55.4.498

[3] Hara H, Harada K, Sukamoto T (1993) Chronological atrophy after transient middle cerebral artery occlusion in rats. Brain Res 618: 251–60. 8374756 10.1016/0006-8993(93)91273-u

[4] McNiece IK, Briddell RA (1995) Stem cell factor. J Leukoc Biol 58: 14–22. 7542304 10.1002/jlb.58.1.14

[5] Greenbaum AM, Link DC (2011) Mechanisms of G-CSF-mediated hematopoietic stem and progenitor mobilization. Leukemia 25: 211–217. 21079612 10.1038/leu.2010.248

[6] Hirata T, Morii E, Morimoto M, Kasugai T, Tsujimura T, et al (1993) Stem cell factor induces outgrowth of c-kit-positive neurites and supports the survival of c-kit-positive neurons in dorsal root ganglia of mouse embryos. Development 119: 49–56. 7506140 10.1242/dev.119.1.49

[7] Motro B, Wojtowicz JM, Bernstein A, van der Kooy D (1996) Steel mutant mice are deficient in hippocampal learning but not long-term potentiation. Proc Natl Acad Sci U S A 93: 1808–1813. 8700840 10.1073/pnas.93.5.1808 PMC39863

[8] Katafuchi T, Li AJ, Hirota S, Kitamura Y, Hori T (2000) Impairment of spatial learning and hippocampal synaptic potentiation in c-kit mutant rats. Learn Mem 7: 383–392. 11112797 10.1101/lm.33900 PMC311355

[9] Diederich K, Sevimli S, Dörr H, Kösters E, Hoppen M, et al (2009) The role of granulocyte-colony stimulating factor (G-CSF) in the healthy brain: a characterization of G-CSF-deficient mice. J Neurosci 29: 11572–11581. 19759304 10.1523/JNEUROSCI.0453-09.2009 PMC6665757

[10] Zhao LR, Navalitloha Y, Singhal S, Mehta J, Piao CS, et al (2007) Hematopoietic growth factors pass through the blood-brain barrier in intact rats. Exp Neurol 204: 569–573. 17307165 10.1016/j.expneurol.2006.12.001 PMC3099460

[11] Zhao LR, Berra HH, Duan WM, Singhal S, Mehta J, et al (2007) Beneficial effects of hematopoietic growth factor therapy in chronic ischemic stroke in rats. Stroke 38: 2804–2811. 17761920 10.1161/STROKEAHA.107.486217

[12] Nudo RJ (2003) Adaptive plasticity in motor cortex: Implications for rehabilitation after brain injury. J Rehabil Med 41 Suppl: 7–10 10.1080/16501960310010070 12817650

[13] Tackenberg C, Ghori A, Brandt R (2009) Thin, stubby or mushroom: spine pathology in Alzheimer's disease. Curr Alzheimer Res 6: 261–268. 19519307 10.2174/156720509788486554

[14] Brown CE, Li P, Boyd JD, Delaney KR, Murphy TH (2007) Extensive turnover of dendritic spines and vascular remodeling in cortical tissues recovering from stroke. J Neurosci 27: 4101–4109. 17428988 10.1523/JNEUROSCI.4295-06.2007 PMC6672555

[15] Mostany R, Chowdhury TG, Johnston DG, Portonovo SA, Carmichael ST, et al (2010) Local hemodynamics dictate long-term dendritic plasticity in peri-infarct cortex. J Neurosci 30: 14116–14126. 20962232 10.1523/JNEUROSCI.3908-10.2010 PMC6634780

[16] Sigler A, Murphy TH (2010) In vivo 2-photon imaging of fine structure in the rodent brain: before, during, and after stroke. Stroke 41: S117–23. 20876484 10.1161/STROKEAHA.110.594648

[17] Feng G, Mellor RH, Bernstein M, Keller-Peck C, Nguyen QT, et al (2000) Imaging neuronal subsets in transgenic mice expressing multiple spectral variants of GFP. Neuron 28: 41–51. 11086982 10.1016/s0896-6273(00)00084-2

[18] Grutzendler J, Kasthuri N, Gan WB (2002) Long-term dendritic spine stability in the adult cortex. Nature 420: 812–816. 12490949 10.1038/nature01276

[19] Tackenberg C, Brandt R (2009) Divergent pathways mediate spine alterations and cell death induced by amyloid-beta, wild-typetau, and R406W tau. J Neurosci 29: 14439. 19923278 10.1523/JNEUROSCI.3590-09.2009 PMC6665808

[20] Neigh GN, Glasper ER, Kofler J, Traystman RJ, Mervis RF, et al (2004) Cardiac arrest with cardiopulmonary resuscitation reduces dendritic spine density in CA1 pyramidal cells and selectively alters acquisition of spatial memory. Eur J Neurosci 20: 1865–1872. 15380008 10.1111/j.1460-9568.2004.03649.x

[21] van Spronsen M, Hoogenraad CC (2010) Synapse pathology in psychiatric and neurologic disease. Curr Neurol Neurosci Rep 10: 207–214. 20425036 10.1007/s11910-010-0104-8 PMC2857788

[22] Bourne JN, Sorra KE, Hurlburt J, Harris KM (2007) Polyribosomes are increased in spines of CA1 dendrites 2 h after the induction of LTP in mature rat hippocampal slices. Hippocampus 17: 1–4. 17094086 10.1002/hipo.20238

[23] Bats C, Groc L (2007) Choquet (2007) The interaction between Stargazin and PSD-95 regulates AMPA receptor surface trafficking. Neuron 53: 719–734. 17329211 10.1016/j.neuron.2007.01.030

[24] Benowitz LI, Carmichael ST (2010) Promoting axonal rewiring to improve outcome after stroke. Neurobiol Dis 37: 259–266. 19931616 10.1016/j.nbd.2009.11.009 PMC2818530

[25] Harris KM, Jensen FE, Tsao B (1992) Three-dimensional structure of dendritic spines and synapses in rat hippocampus (CA1) at postnatal day 15 and adult ages: implications for the maturation of synaptic physiology and long-term potentiation. J Neurosci 12: 2685–705. 1613552 10.1523/JNEUROSCI.12-07-02685.1992 PMC6575840

[26] Matsuzaki M, Ellis-Davies GC, Nemoto T, Miyashita Y, Iino M, et al (2001) Dendritic spine geometry is critical for AMPA receptor expression in hippocampal CA1 pyramidal neurons. Nat Neurosci 4: 1086–1092. 11687814 10.1038/nn736 PMC4229049

[27] Spacek J, Harris KM (1997) Three-dimensional organization of smooth endoplasmic reticulum in hippocampal CA1 dendrites and dendritic spines of the immature and mature rat. J Neurosci 17: 190–203. 8987748 10.1523/JNEUROSCI.17-01-00190.1997 PMC6793680

[28] Cooney JR, Hurlburt JL, Selig DK, Harris KM, Fiala JC (2002) Endosomal compartments serve multiple hippocampal dendritic spines from a widespread rather than a local store of recycling membrane. J Neurosci 22: 2215–2224. 11896161 10.1523/JNEUROSCI.22-06-02215.2002 PMC6758269

[29] Witcher MR, Kirov SA, Harris KM (2007) Plasticity of perisynaptic astroglia during synaptogenesis in the mature rat hippocampus. Glia 55: 13–23. 17001633 10.1002/glia.20415

[30] Bourne J, Harris KM (2007) Do thin spines learn to be mushroom spines that remember? Curr Opin Neurobiol 17: 381–386. 17498943 10.1016/j.conb.2007.04.009

[31] O'Brien RJ, Lau LF, Huganir RL (1998) Molecular mechanisms of glutamate receptor clustering at excitatory synapses. Curr Opin Neurobiol 8: 364–369. 9687358 10.1016/s0959-4388(98)80062-7

[32] Xu W (2011) PSD-95-like membrane associated guanylate kinases (PSD-MAGUKs) and synaptic plasticity. Curr Opin Neurobiol 21: 306–312. 21450454 10.1016/j.conb.2011.03.001 PMC3138136

[33] Béïque JC, Lin DT, Kang MG, Aizawa H, Takamiya K, et al (2006) Synapse-specific regulation of AMPA receptor function by PSD-95. Proc Natl Acad Sci U S A 103: 19535–19540. 17148601 10.1073/pnas.0608492103 PMC1748260

[34] Chen X, Leischner U, Rochefort NL, Nelken I, Konnerth A (2011) Functional mapping of single spines in cortical neurons in vivo. Nature 475: 501–505. 21706031 10.1038/nature10193

[35] Noguchi J, Nagaoka A, Watanabe S, Ellis-Davies GC, Kitamura K, et al (2011) In vivo two-photon uncaging of glutamate revealing the structure-function relationships of dendritic spines in the neocortex of adult mice. J Physiol 589: 2447–2457. 21486811 10.1113/jphysiol.2011.207100 PMC3115818

[36] Brown CE, Boyd JD, Murphy TH (2010) Longitudinal in vivo imaging reveals balanced and branch-specific remodeling of mature cortical pyramidal dendritic arbors after stroke. J Cereb Blood Flow Metab 30: 783–791. 19920846 10.1038/jcbfm.2009.241 PMC2949167

[37] Mostany R, Portera-Cailliau C (2011) Absence of large-scale dendritic plasticity of layer 5 pyramidal neurons in peri-infarct cortex. J Neurosci 31: 1734–1738. 21289182 10.1523/JNEUROSCI.4386-10.2011 PMC6623721

[38] Chapleau CA, Carlo ME, Larimore JL, Pozzo-Miller L (2008) The actions of BDNF on dendritic spine density and morphology in organotypic slice cultures depend on the presence of serum in culture media. J Neurosci Methods 169: 182–190. 18242714 10.1016/j.jneumeth.2007.12.006 PMC2348185

[39] Murphy TH, Corbett D (2009) Plasticity during stroke recovery: from synapse to behaviour. Nat Rev Neurosci 10: 861–872. 19888284 10.1038/nrn2735

[40] Biernaskie J, Chernenko G, Corbett D (2004) Efficacy of rehabilitative experience declines with time after focal ischemic brain injury. J Neurosci 24: 1245–1254. 14762143 10.1523/JNEUROSCI.3834-03.2004 PMC6793570

