# Supplementary figures and images for: Reestablishing Neuronal Networks in the Aged Brain by Stem Cell Factor and Granulocyte-Colony Stimulating Factor in a Mouse Model of Chronic Stroke
Source: PLoS One. 2013 Jun 4;8(6):e64684. doi: 10.1371/journal.pone.0064684 (PMC3672166; doi:10.1371/journal.pone.0064684)

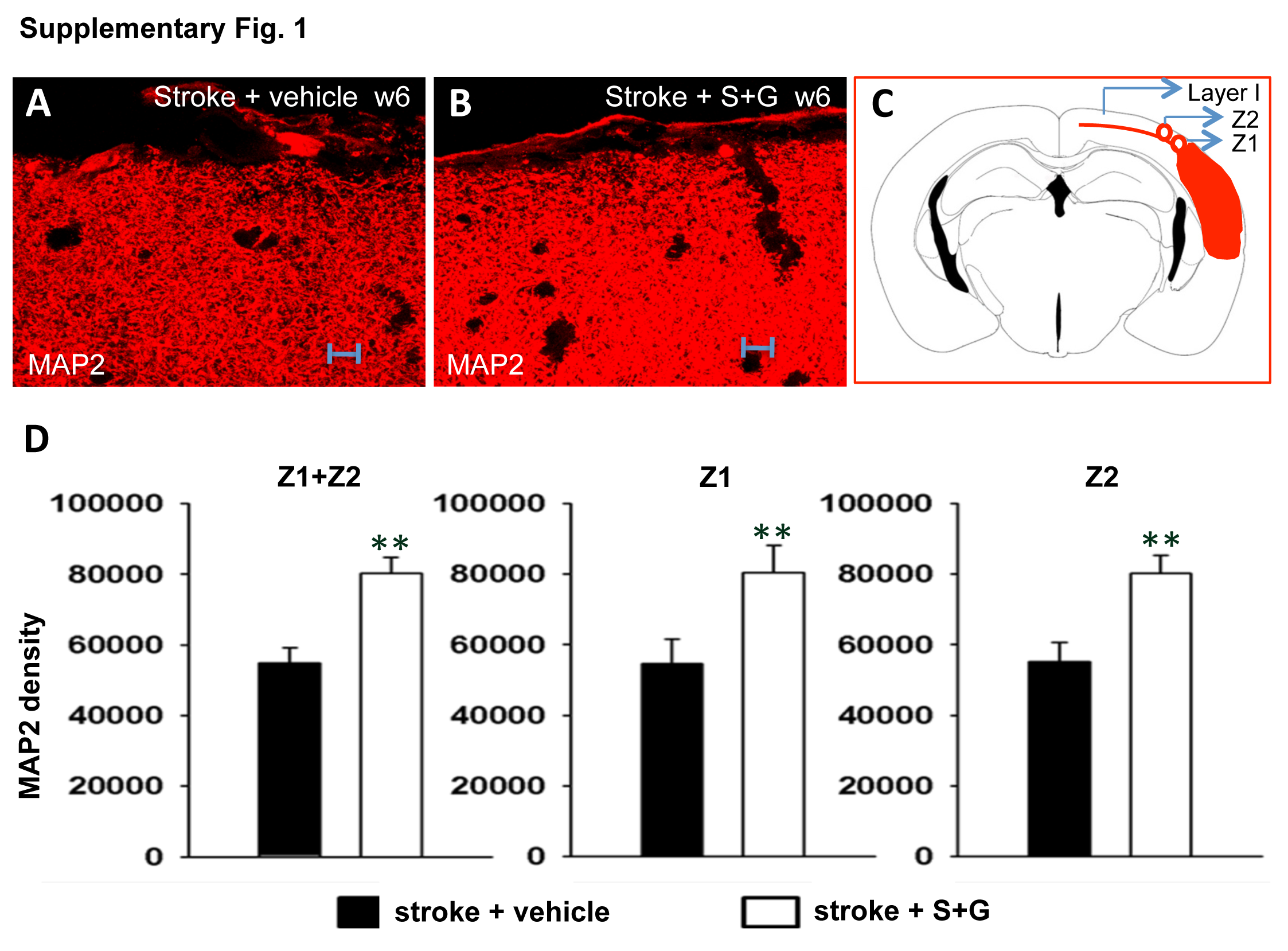

Supplement: Figure S1 — SCF+G-CSF treatment in chronic stroke increases dendritic density in the parietal cortex outside the infarct cavities 6 weeks after treatment in the aged brain. (A and B) Representative images of 3-dimensional projection of MAP2-labeled dendrites in the brain of stroke with vehicle treatment (A) and stroke with SCF+G-CSF treatment (B). (C) A schematic graph shows where the images were taken in the parietal cortex. (D) Quantification of dendritic density in the layer I cortex surrounding the infarct cavities. Scale bar: 50 µm. Z1, zone 1. Z2, zone 2. **p<0.01. N = 3–4. Mean ± S.E.M. (TIF) [file pone.0064684.s001.tif]

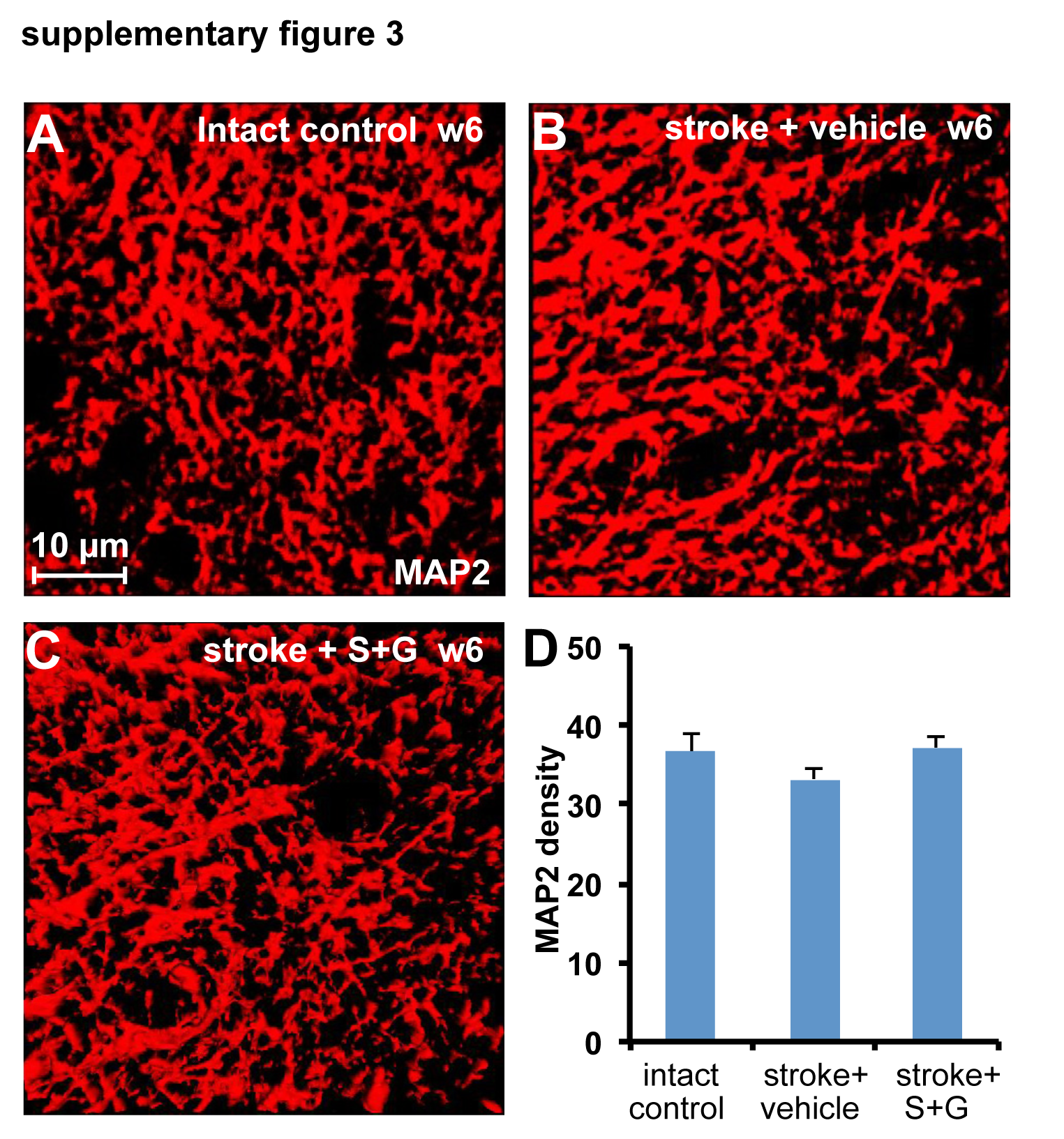

Supplement: Figure S2 — SCF+G-CSF treatment in chronic stroke does not affect the dendritic density in the contralateral cortex of the aged brain. (A–C) Representative images of 3-dimensional projection of MAP2-labeled dendrites in the contralateral cortex (layer I) of an intact brain (A), the stroke brains treated with vehicle (B) or SCF+G-CSF (S+G) (C) 6 weeks after treatment. (D) Quantification of dendritic density in the layer I cortex contralateral to the infarct brain. N = 3–6. Mean ± S.E.M. (TIF) [file pone.0064684.s002.tif]
